# Barriers to sealant guideline implementation within a multi-site managed care dental practice

**DOI:** 10.1186/s12903-018-0480-z

**Published:** 2018-02-02

**Authors:** Deborah E. Polk, Robert J. Weyant, Nilesh H. Shah, Jeffrey L. Fellows, Daniel J. Pihlstrom, Julie Frantsve-Hawley

**Affiliations:** 1 University of Pittsburgh School of Dental Medicine, 3501 Terrace Street, 381 Salk Hall, Pittsburgh, PA 15261 USA; 2 University of Pittsburgh School of Dental Medicine, 3501 Terrace Street, 347 Salk Hall, Pittsburgh, PA 15261 USA; 3 University of Pittsburgh School of Dental Medicine, 3501 Terrace Street, 389 Salk Hall, Pittsburgh, PA 15261 USA; 4 Kaiser Permanente Center for Health Research, 3800 N Interstate Ave., Portland, OR 97227-1110 USA; 5 Permanente Dental Associates, 500 NE Multnomah St., Suite 100, Portland, OR 97232 USA; 6 Julie Frantsve-Hawley Consulting, 621 Brier Street, Kenilworth, IL 60043-0000 USA

**Keywords:** Evidence-based dentistry, Guideline implementation, Noncavitated carious lesions, Barriers, Implementation strategies, Multi-site dental group practice, Pit-and-fissure sealants, Incipient caries, Caries management

## Abstract

**Background:**

The purpose of this study was to identify barriers frequently endorsed by dentists in a large, multi-site dental practice to implementing the American Dental Association’s recommendation for sealing noncavitated occlusal carious lesions as established in their 2016 pit-and-fissure sealant clinical practice guideline. Although previous research has identified barriers to using sealants perceived by dentists in private practice, barriers frequently endorsed by dentists in large, multi-site dental practices have yet to be identified. Identifying barriers for these dentists is important, because it is expected that in the future, the multi-site group practice configuration will comprise more dental practices.

**Methods:**

We anonymously surveyed the 110 general and pediatric dentists at a multi-site dental practice in the U.S. The survey assessed potential barriers in three domains: practice environment, prevailing opinion, and knowledge and attitudes. Results were summarized using descriptive statistics.

**Results:**

The response rate to the survey was 62%. The principal barrier characterizing the practice environment was concern regarding liability; endorsed by 33% of the dentists. Many barriers of prevailing opinion were frequently endorsed. These included misunderstanding the standard of practice (59%), being unaware of the expectations of opinion leaders (56%) including being unaware of the guideline itself (67%), and being unaware of what is currently being taught in dental schools (58%). Finally, barriers of knowledge and attitudes were frequently endorsed. These included having suboptimal skill in applying sealants (23% - 47%) and lacking knowledge regarding the relative efficacy of the different ways to manage noncavitated occlusal carious lesions (50%).

**Conclusions:**

We identified barriers frequently endorsed by dentists in a large, multi-site dental practice relating to the practice environment, prevailing opinion, and knowledge and attitudes. All the barriers we identified have the potential to be addressed by implementation strategies. Future studies should devise and test implementation strategies to target these barriers.

**Electronic supplementary material:**

The online version of this article (10.1186/s12903-018-0480-z) contains supplementary material, which is available to authorized users.

## Background

Dental caries in young children is a significant public health concern in the U.S. In the U.S. in 2011–2012, 55.7% of children aged 6–8 years had caries experience in their primary teeth, and 28.8% of children aged 9–11 years had caries experience in their permanent teeth [1]. The occlusal surfaces of the molars are the most vulnerable to caries, with the probability of developing caries in these teeth among 5- to 16-year-old children ranging from 0.23 to 0.34 over four years [2]. The most effective way to protect these surfaces is with dental sealants. Not only do sealants prevent the onset of new caries, but they can also arrest the progression of early caries, preventing the need for a restoration. A recent, systematic review concluded that compared with children and adolescents who did not receive sealants, children and adolescents who received sealants on sound occlusal surfaces or on early caries in their primary or permanent molars experienced a 76% reduction in the risk of developing new carious lesions over the subsequent two years [3]. The effectiveness of sealants on occlusal surfaces far exceeds the benefits provided by commonly used interventions such as topical fluoride applications and professional dental cleanings [4]. Consequently, in 2008 the American Dental Association (ADA) developed, and in 2016 updated, the “Evidence-based clinical practice guideline for the use of pit-and-fissure sealants” [5, 6]. Despite the comprehensive dissemination of the ADA’s guideline, estimated compliance among general dentists ranges from 0 to 5% [7] to 38.5% to 50%, [8] representing a significant quality and implementation gap.

Currently, the literature identifying barriers to implementing the pit-and-fissure sealant guideline is limited to just two studies [7, 9]. Barriers include dentists being unaware of the guideline; [7] not knowing the difference between an early lesion that can be arrested with a sealant versus a more established lesion that must receive a restoration; [7] not believing in the effectiveness of sealants to arrest decay; [7] not having the office workflow structure to support the application of sealants; [9] not believing that applying sealants is the standard of care; [7, 9] and not finding incentive in the current reimbursement structure [9]. Theory suggests that barriers to change are setting-specific and must be identified for the specific setting in which one desires to make change [10]. In both of these studies, the samples were composed primarily of dentists in private practice. Although private practices and large, multi-site managed care practices are similar in many respects, there are important differences, such as how practice policies are set, that could affect implementation.

Although most U.S. dentists continue to practice in solo and small group practices, large, multi-site group practices are becoming more common in dentistry. From 2002 to 2012, the ADA Health Policy Institute’s analysis of U.S. Census Bureau data showed that the percentage of total U.S. dental care revenue from dental practices with 20 or more employees grew from 15.7% to 20.1%, whereas for dental practices with fewer than five employees, the percentage of total receipts fell from 19.9% to 16.0% [11]. Similar to general medical care, economic factors have fostered a transition from solo and small group practices to large group practices. The ADA predicts consolidation and multi-group practices will increase substantially in the coming years [12]. Thus, this trend supports the value and importance of studying implementation within large group practices.

The purpose of the present study is to identify barriers frequently endorsed by dentists in a large, multi-site dental practice to implementing the ADA’s pit-and-fissure sealant guideline. We hypothesize that dentists are unaware of the guideline; they do not know the difference between an early lesion that can be arrested with a sealant versus a more established lesion that must receive a restoration; they do not believe in the effectiveness of sealants to arrest decay; they do not have the office workflow structure to support the application of sealants; and they do not believe that applying sealants is the standard of care.

## Methods

In fall, 2016, we anonymously surveyed all 110 general and pediatric dentists at a multi-site dental practice in the U.S. The study team used the practice’s internal email system to recruit survey participants. The email invitation explained the purpose of the study, encouraged participation, and provided a link to anonymous Web-based survey. Email reminders to complete the survey were sent after one and two weeks. The survey was administered via Qualtrics (Qualtrics, Provo, Utah). The Kaiser Permanente Northwest Institutional Review Board reviewed and approved this study (STUDY00000636).

We developed items for the survey using a combination of strategies. To generate a list of barriers faced by dentists, we relied on an existing classification of types of barriers [13]. From this classification, we focused on barriers in three domains: practice environment (organizational context), prevailing opinion (social context), and knowledge and attitudes (professional context). Questions assessing the practice environment addressed financial disincentives, organizational constraints such as the workflow, perception of liability, and patients’ expectations. Because we assumed that the practice environment would be similar for placing sealants for preventing caries and for arresting NCCL, we used questions about practice environment barriers to placing sealants for preventing caries to learn about practice environment barriers to placing sealants for arresting NCCL. Questions assessing the prevailing opinion addressed standards of practice, opinion leaders, training, awareness of the guideline, and awareness of practice policy. Questions assessing knowledge and attitudes addressed sense of competence, the perceived need to do something, and beliefs regarding effective treatments. We also reviewed items from previous surveys [7–9]. Then, in an iterative process, we generated items for each type of barrier and obtained feedback on the items from clinical and non-clinical employees of the dental practice from which we were administering the survey until we had a set of items that clearly captured each issue. Examples and the type of barrier assessed include the following: “Placing sealants to arrest the progression of noncavitated carious lesions would put me at risk from a liability perspective” (practice environment); “Do you think that placing sealants to arrest the progression of noncavitated carious lesions is within the standard of care” (prevailing opinion); and “Do you think restoring a noncavitated occlusal carious lesion provides a better outcome for the patient when compared with a sealant” (knowledge and attitudes). For all questions, the response set was multiple choice, though the particular responses varied depending on the question (see Addititonal file 1 for the complete text of the survey).

Descriptive statistics were calculated using SAS Enterprise Guide Version 5.1 (SAS Institute Inc., Cary, NC). Some dentists skipped some of the questions. Missing data were assumed to be missing completely at random.

## Results

Of the 110 general or pediatric dentists in the practice, 63 general dentists and 5 pediatric dentists responded to the survey for a response rate of 62%. Of the 44 dentists who answered the question about their year of graduation from dental school, the year ranged from 1981 to 2012, with the average being 1999 (SD = 9.5) and the median being 2003. The sample range is slightly constricted compared with the range for all PDA general and pediatric dentists, 1976–2016; the sample average is slightly older compared with the PDA population average (2001); and the median is the same.

### Practice environment (organizational context)

The only practice environment barrier to placing sealants on NCCLs endorsed by many dentists was being put at risk from a liability perspective (Table 1). Potential barriers that were not endorsed by many dentists included having a work environment that was not conducive to placing sealants and having to manage patient complaints when sealants needed to be replaced (Table 1).

**Table 1:** Percentage of dentists endorsing barriers in the practice environment to placing sealants on NCCL and potential implementation strategies targeting barriers in the practice environment

Question	Response option 1	Response option 2	Response option 3	Response option 4	Response option 5
	Percentage (n/total responding)	Percentage (n/total responding)	Percentage (n/total responding)	Percentage (n/total responding)	Percentage (n/total responding)
Potential Barriers					
Placing sealants to arrest the progression of NCCL would put me at risk from a liability perspective.	Strongly agree 2% (1/65)	Somewhat agree 32% (21/65)	Neither agree nor disagree 29% (19/65)	Somewhat disagree 26% (17/65)	Strongly disagree 26% (17/65)
How often do you place sealants to prevent occlusal caries?	Always 36% (24/67)	Frequently 58% (39/67)	Occasionally 6% (4/67)	Rarely 0%	Never 0%
How often do patients complain when a sealant needs to be replaced?	Always 0%	Frequently 0%	Occasionally 9% (6/67)	Rarely 49% (3/67)	Never 42% (28/67)
Implementation Strategies					
Could hygiene and assistant staff do an adequate job placing a sealant over an NCCL?	Yes 35% (23/65)	Maybe 46% (30/65)	No 18% (12/65)		
Does the hygiene and assistant staff have capacity in their schedules to treat patients who need sealants for NCCL?	Yes 11% (7/65)	Maybe 55% (36/65)	No 34% (22)		
How easy would it be to change the workflow in your clinic to allow the dental hygienist and dentist to routinely apply sealants on NCCL?	Extremely easy 2% (1/65)	Somewhat easy 14% (9/65)	Neither easy nor difficult 25% (16/65)	Somewhat difficult 45% (29/65)	Extremely difficult 15% (10/65)

If the practice environment had not been conducive to placing sealants, one potential implementation strategy would have been to have the dental hygiene and assistant staff place the sealants. Respondent opinions regarding this strategy were mixed (Table 1). Although many dentists believed that hygiene and assistant staff could do an adequate job placing a sealant, many dentists believed that the hygiene and assistant staff do not have the capacity in their schedules to place sealants; and they believed it would not be easy to change the workflow to allow the dentist and dental hygienist to place sealants.

### Prevailing opinion (social context)

Prevailing opinion barriers to placing sealants on NCCLs endorsed by many dentists included underestimating the percentage of their colleagues who were already implementing the guideline; being unaware of the expectations of opinion leaders, including being unaware of the guideline itself and that applying sealants to NCCL was official practice policy; and; misunderstanding the standard of practice (Table 2). All the prevailing opinion barriers we asked about were endorsed by many dentists.

**Table 2:** Percentage of dentists endorsing barriers of prevailing opinion to placing sealants on NCCL and potential implementation strategies targeting barriers of prevailing opinion

Question	Response option 1 Percentage (n/total responding)	Response option 2 Percentage (n/total responding)	Response option 3 Percentage (n/total responding)	Response option 4 Percentage (n/total responding)	Response option 5 Percentage (n/total responding)
Potential Barriers					
What percentage of your PDA colleagues do you believe routinely apply sealants to NCCL?	0–5% 17% (1/65)	6–25% 37% (24/65)	26–50% 15% (10/65)	More than 50% 31% (20/65)	
Are you aware of any guidelines published by a professional dental organization or society (outside of PDA) regarding the use of sealants to manage NCCL?	Yes 34% (22/65)	No 66% (43/65)			
Do you think that placing sealants to arrest the progression of NCCLs is within the standard of care?	Yes 42% (27/65)	Maybe 45% (29/65)	No 14% (9/65)		
Implementation Strategies					
Are you aware of any guidance/policy from the PDA CEC regarding how NCCL should be managed?	Yes 45% (29/65)	No 55% (36/65)			
Asked of those who answered “Yes” above: Do you think having guidance/policy from the PDA CEC on managing NCCL is a good thing?	Yes 83% (24/29)	Maybe 14% (4/29)	No 3% (1/29)		
Asked of those who answered “No” above: Do you think the PDA CEC should provide guidance/policy regarding how NCCL should be managed?	Yes 61% (22/36)	Maybe 28% (10/36)	No 11% (4/36)		
Do you think the PDA CEC should establish clinical performance standards regarding how NCCL should be managed?	Yes 45% (29/65)	Maybe 42% (27/65)	No 14% (9/65)		
Do you think the PDA CEC should conduct audits and provide individual performance feedback regarding how you manage NCC?	Yes 18% (12/65)	Maybe 48% (31/65)	No 34% (22/65)		
Is part of your salary based on quality measures regarding how you manage your patients?	Yes 86% (56/65)	Maybe 11% (7/65)	No 3% (2/65)		

**Fig. 1:**
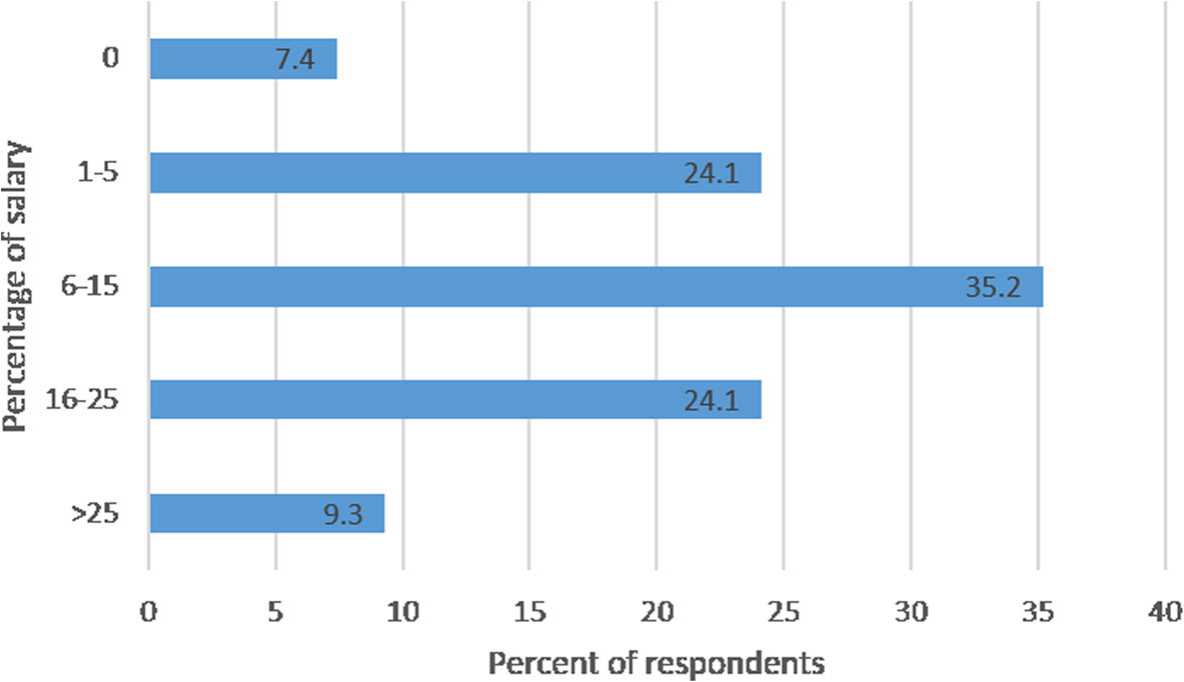
Percentage of respondents endorsing the percentage of their salary to be based on patient management

An implementation strategy targeting the dentists’ lack of awareness of the guideline and standard of care could be for the practice to establish policy or clinical performance standards regarding how NCCL should be managed. Generally, these strategies were supported by the dentists (Table 2). In fact, the practice had already implemented a policy; however, as described above, many of the dentists were unaware of it. An implementation strategy targeting the dentists’ lack of awareness of their practice’s policy could be for the practice to conduct audits and provide individual performance feedback regarding how the dentists manage NCCL. This approach was not strongly endorsed by the dentists (Table 2). A different implementation strategy targeting the same barrier could be for the practice to provide a financial incentive for applying sealants. Currently, the practice does provide incentives for performance, including applying sealants, and, in fact, many of the dentists were aware that part of their salary is based on quality measures regarding how they manage their patients (Table 2). Given the current lack of adoption of sealing NCCL, however, it appears that the current percentage is not sufficient to change behavior. The dentists had a range of opinions regarding the percentage of their salary they thought should be based on how they manage their patients (Fig. 1).

### Knowledge and attitudes (professional context)

**Fig. 2:**
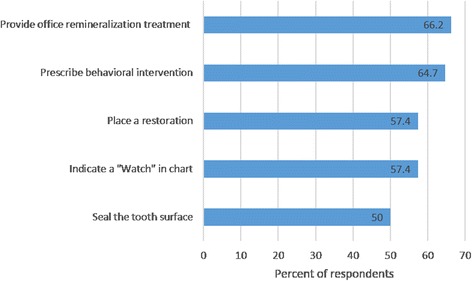
Percentage of respondents endorsing approaches they adopt when treating NCCL. Percentages sum to great than 100% because respondents were allowed to select as many response options as applied

Knowledge and attitude barriers to placing sealants on NCCLs endorsed by many dentists included lacking knowledge regarding the relative efficacy of the different ways to manage NCCL (Fig. 2); believing that sealants are not effective in arresting decay; believing that restoring an NCCL provides a better outcome than sealing; and having suboptimal skill in applying sealants (Table 3). Potential barriers that were not endorsed by many dentists included lacking familiarity with the ADA caries classification system definitions of noncavitated, initial caries, and cavitated carious lesions [14] that we provided in the survey and failing to distinguish between lesions with and without macroscopic breakdown in surface tooth structure (i.e., cavitated versus noncavitated) when diagnosing carious occlusal lesions (Table 3).

**Table 3:** Percentage of dentists endorsing barriers of knowledge and attitudes to placing sealants on NCCL and potential implementation strategies targeting barriers of knowledge and attitudes

Question	Response option 1 Percentage (n/total responding)	Response option 2 Percentage (n/total responding)	Response option 3 Percentage (n/total responding)	Response option 4 Percentage (n/total responding)	Response option 5 Percentage (n/total responding)
Potential Barriers					
Sealants are very effective in arresting decay when there is noncavitated occlusal caries.	Strongly agree 18% (12/66)	Somewhat agree 27% (18/66)	Neither agree nor disagree 27% (18/66)	Somewhat disagree 24% (16/66)	Strongly disagree 3% (2/66)
Do you think restoring a noncavitated occlusal carious lesion provides a better outcome for the patient when compared with a sealant?	Always 9% (6/65)	Most of the time 28% (18/65)	About half the time 14% (9/65)	Sometimes 43% (28/65)	Never 6% (4/65)
Among your patients, how well do sealants prevent occlusal caries?	Extremely well 30% (20/67)	Very well 48% (32/67)	Moderately well 21% (14/67)	Slightly well 1% (1/67)	Not well at all 0%
Among your patients, how often do sealants fail (i.e., caries occurs) within a year?	Frequently 3% (2/67)	Occasionally 43% (29/67)	Rarely 52% (35/67)	Never 1% (1/67)	
Among your patients, for how long do sealants last before ultimately needing to be touched up or replaced?	6–12 months 2% (1/66)	13–24 months 29% (19/66)	25–36 months 48% (32/66)	More than 36 months 21% (14/66)	
How familiar were you with these definitions prior to starting the survey?	Extremely familiar 30% (20/67)	Very familiar 36% (24/67)	Moderately familiar 27% (18/67)	Slightly familiar 6% (4/67)	Not familiar at all 1% (1/67)
In general, when you diagnose carious occlusal lesions, do you distinguish between lesions with and without macroscopic breakdown in surface tooth structure (i.e., cavitated versus noncavitated) as defined above?	Yes 70% (46/66)	No 30% (20/66)			
Implementation Strategies					
Have you heard about the development of diagnostic codes in dentistry?	Yes 55% (36/65)	No 45% (29/65)			

If failing to distinguish between lesions with and without macroscopic breakdown had been a barrier, one potential implementation strategy would have been to introduce diagnostic codes (Table 3).

## Discussion

The purpose of this study was to identify barriers frequently endorsed by salaried dentists in a multi-site practice in implementing the ADA’s pit-and-fissure sealant guideline. Because multi-site practices are structurally different from small private practices, the barriers to implementing guidelines may be different. Based on the results of the survey, we identified barriers in three broad domains: the practice environment; prevailing opinion; and knowledge and attitudes. Consistent with our hypotheses, we found that dentists were unaware of the guideline; they did not believe in the effectiveness of sealants to arrest decay; and they did not believe that applying sealants is the standard of care. Contrary to hypothesis, the dentists did know the difference between an early lesion that can be arrested with a sealant versus a more established lesion that must receive a restoration; and they did have the office workflow structure to support the application of sealants.

Some of the barriers identified were related to the practice environment. For example, over 33% of the dentists “somewhat” or “strongly” agreed with the idea that placing sealants to arrest the progression of NCCL would put them at risk from a liability perspective. Thus, although not identified in either of the previous studies, [7, 9] concerns about liability risk may be a barrier. Liability is closely related to the standard of care, which is a barrier relating to the prevailing opinion. It is when the dentist practices below the standard of care that he or she is subject to liability for injury or damage to the patient, or legally, for tortious conduct [15]. Slightly more than half the dentists in the survey (58%) thought that placing sealants may be or is not within the standard of care. It may be that the dentists were relying on an out-of-date understanding of how the standard of care is determined in their state. Previously the standard of care was based on the locality, or Frye, rule, which defined it by general acceptance among local experts [16]. Consistent with the possibility that the dentists were relying on the Frye rule, most of the dentists we surveyed underestimated the percentage of their colleagues who were, in fact, sealing over NCCL. Starting 1993, however, the way the standard of care is determined began to change; in the state in which this study was conducted and in many other states, it is now based on the Daubert rule [16]. According to the Daubert rule, the standard of care is based on scientific evidence deemed acceptable by a judge [16]. Thus, to address the dentists’ liability concerns, dentists may need to be educated regarding how the standard of care is determined and what the evidence is.

In addition to concerns about liability, there are other aspects of the practice environment, such as the workflow. One previous study identified the workflow as a barrier, [9] however, that study was not focused specifically on the pit-and-fissure guideline. Given that over 90% of the dentists are placing sealants to prevent caries, workflow does not appear to be a barrier.

Some of the barriers identified were related to prevailing opinion. For example, less than half the dentists believed that placing sealants to arrest the progression of NCCL was within the standard of care. This finding is consistent with both the previous studies [7, 9] and with the dentists’ perception of the social norm, as described above. As discussed above, it may be possible to address this barrier by educating dentists about how the standard of care is determined in their state and the evidence on which it is based. It may also be useful to enhance the ways the dentists communicate with each other about their approaches to treatment.

Another barrier reflecting prevailing opinion is lack of awareness of the guideline. Consistent with findings from the literature, [7] only one third of the dentists surveyed reported being aware of the guideline. Furthermore, less than half the dentists were aware that their practice had issued guidance regarding how NCCL should be managed. Thus, there appears to be a barrier in communicating expectations, at both the national and practice levels. Given that current approaches are not sufficient, we may need to query dentists to find out how best to disseminate information to them. Anecdotal evidence suggests that dentists turn to trusted colleagues for information [7]. We may need to identify ways to maximize this communication channel. Based on these results, it appears that multiple sources are failing to provide dentists information regarding the ADA’s pit-and-fissure guideline. Thus, it is no wonder that they do not realize it is the standard of care. Furthermore, ineffective communication may be a consistent problem across barriers having to do with the prevailing opinion. Problems in communication appeared to be apparent in all aspects of these barriers.

Finally, some of the barriers identified were related to knowledge and attitudes. For example, based on the suboptimal performance of the sealants the dentists apply for prevention of caries, they may need additional training in the application of sealants. Also, given that the dentists adopt less efficacious over more efficacious approaches to managing NCCL, they appear to be lacking information about the relative efficacy of the different approaches. This is consistent with previous research [7] And given the dentists’ inclusion of fissurotomy when they do apply sealants, they may not understand the mechanism of action of sealants. These barriers can be addressed through training and education. One factor that does not appear to be a barrier is the dentists’ familiarity with the definitions of noncavitated, initial caries, and cavitated lesions. This finding differs from previous research [7]. Furthermore, consistent with their self-report of familiarity with the definitions, when making diagnoses, most of the dentists report distinguishing between cavitated and noncavitated carious lesions. Additionally, they have favorable attitudes toward the ability of sealants to prevent caries. This may be a starting point on which to build favorable attitudes toward the ability of sealants to arrest incipient decay. Thus, barriers relating to knowledge and attitudes are present and need to be addressed by implementation strategies.

There were several strengths of this study. To our knowledge, we are the first to examine barriers to implementing the ADA’s pit-and-fissure guideline in a large, multi-site dental practice. In addition, because we followed a comprehensive classification scheme [13] when designing the survey questions, the content validity of the survey instrument was strengthened. Limitations of the study include unknown reliability of the survey instrument and the possibility of non-responder bias. We attempted to address the possibility of non-responder bias by pilot testing the survey instrument with employees of the dental practice [17]. Because the survey was anonymous, however, we have no way to determine whether the dentists who did not respond to the survey are different in relevant ways from the ones who did [17].

In sum, we surveyed general and pediatric dentists in a large, multi-site dental practice to identify barriers they face in implementing the ADA’s pit-and-fissure sealant guideline. We identified barriers relating to the practice environment, prevailing opinion, and knowledge and attitudes. Some of the barriers frequently endorsed in our study, such as being unaware of the guideline, not believing in the effectiveness of sealants to arrest decay, and not believing that applying sealants to arrest NCCL is the standard of care had been identified in previous studies, while others were new to this study. Barriers not mentioned in previous studies included concerns about risk from a liability perspective, perceptions of the social norm, poor technique in applying sealants, and misperceptions regarding the efficacy of sealants relative to other treatment approaches. This is consistent with theory, which suggests that barriers are setting-specific [10]. All the barriers we identified have the potential to be addressed by implementation strategies. Barriers to implementing guidelines exist at multiple levels of the healthcare system [18, 19]. The focus of this study was on barriers endorsed frequently by dentists. Future studies should address barriers identified by players involved in other levels, as well. Future studies should also address facilitators to implementing guidelines. Once barriers and facilitators are identified, future studies should identify implementation strategies to break down the barriers and strengthen the facilitators. These implementation strategies can then be tested in clinical trials and disseminated.

## Conclusions

We conclude that in this large, multi-site dental practice, there are barriers to implementing the ADA’s guideline on dental sealants. Some of these barriers had ben endorsed in previous studies, but some had not been endorsed before. All these barriers could affect the implementation of the guideline. Knowledge of barriers can help us develop implementation strategies that target these barriers. The implementation strategies developed can be used wherever the barriers are identified, leading to increased implementation of the guideline and improved care for patients.

## Additional file

Additional file 1: Use of Dental Sealants by KP Dentists. This file contains the text, coding, and skip patterns of the survey we administered to the study participants. (DOCX 17 kb)

## Abbreviations

ADA: American Dental Association
NCCL: noncavitated occlusal carious lesion
