# Supplementary material for: Barriers to sealant guideline implementation within a multi-site managed care dental practice
Source: BMC Oral Health. 2018 Feb 2;18:17. doi: 10.1186/s12903-018-0480-z (PMC5797385; doi:10.1186/s12903-018-0480-z)
Supplement: Additional file 1: — Use of Dental Sealants by KP Dentists. This file contains the text, coding, and skip patterns of the survey we administered to the study participants. (DOCX 17 kb) [file 12903_2018_480_MOESM1_ESM.docx]

Use of Dental Sealants by KP Dentists

Q1 What is your primary specialty?

- General Dentistry (1)
- Pediatric Dentistry (2)
- Other (3)

If Other Is Selected, Then Skip To End of Survey

Q2 This survey is being conducted to learn about dentists' knowledge and practice patterns around the management of noncavitated occlusal carious lesions.

Q3 Preventing Caries

Q4 Sealants are very effective in preventing occlusal caries.

- Strongly agree (1)
- Somewhat agree (2)
- Neither agree nor disagree (3)
- Somewhat disagree (4)
- Strongly disagree (5)

Q5 How often do you place sealants to prevent occlusal caries?

- Always (1)
- Frequently (2)
- Occasionally (3)
- Rarely (4)
- Never (5)

If Rarely Is Selected, Then Skip To How familiar were you with these defi...

Q6 Among your patients, how well do sealants prevent occlusal caries?

- Extremely well (1)
- Very well (2)
- Moderately well (3)
- Slightly well (4)
- Not well at all (5)

Q7 Among your patients, how often do sealants fail (i.e., caries occurs) within a year?

- Always (1)
- Frequently (2)
- Occasionally (3)
- Rarely (4)
- Never (5)

Q8 Among your patients, for how long do sealants last before ultimately needing to be touched up or replaced?

- 6 - 12 months (1)
- 13 - 24 months (2)
- 25 - 36 months (3)
- More than 36 months (4)

Q9 How often do patients complain when a sealant needed to be replaced?

- Always (1)
- Frequently (2)
- Occasionally (3)
- Rarely (4)
- Never (5)

Q10 Noncavitated Carious Lesions

This next set of questions addresses noncavitated carious lesions. Please refer to the definitions below when answering the questions.

Noncavitated lesions: are "characterized by a change in color, glossiness, or surface structure as a result of demineralization before there is macroscopic breakdown in surface tooth structure." ADA Caries Classification System (CCS)

Initial caries lesions: are "the earliest detectable lesions compatible with net mineral loss. They are limited to the enamel or cementum or very outermost layer of dentin on the root surface and, in the mildest forms, are detectable only after drying.... In pits and fissures, there is a clear change in color to brown but no sign of significant demineralization in the dentin (that is, no underlying dark gray shadow). These initial lesions are considered noncavitated, and, with remineralization, are reversible." CCS

Cavitated carious lesions: have a "loss of surface integrity. In some cases, cavitation can be restricted to the enamel (for example, microcavitation). ... Frequently, cavitation refers to the total loss of enamel and exposure of the underlying dentin." CSS

Q11 How familiar were you with these definitions prior to starting the survey?

- Extremely familiar (1)
- Very familiar (2)
- Moderately familiar (3)
- Slightly familiar (4)
- Not familiar at all (5)

Q12 In general, when you diagnose carious occlusal lesions, do you distinguish between lesions with and without macroscopic breakdown in surface tooth structure (i.e., cavitated versus noncavitated) as defined above?

- Yes (1)
- No (2)

Q13 How do you generally treat noncavitated occlusal carious lesions? Select as many as apply.

- Indicate a "Watch" in patient chart (1)
- Prescribe a behavioral intervention (e.g., brush more, use fluoride mouth rinse, etc.) (2)
- Provide an office remineralization treatment (e.g., topical fluoride application) (3)
- Seal the tooth surface (4)
- Place a restoration of some type (preventive resin restoration, composite, amalgam) (5)
- Other (please specify): (6) ____________________

Q14 In your practice, how often do you place sealants over noncavitated occlusal carious lesions without altering the occlusal surface with a bur or some other mechanical preparation?

- Always (1)
- Frequently (2)
- Occasionally (3)
- Rarely (4)
- Never (5)

If Rarely Is Selected, Then Skip To Was the option of sealing over non-ca...

Q15 What are the factors that influence whether you decide to place a sealant over a noncavitated occlusal carious lesion? Select as many as apply.

- No or minimal radiographic evidence of caries (1)
- Confidence the patient will comply with future exams (2)
- Confidence the sealant can be placed under good moisture control (3)
- Time in schedule (4)
- Other (please specify): (5) ____________________

Q16 When you place a sealant over a noncavitated occlusal carious lesion, do you typically open the "grooves" (i.e., fissurotomy) before restoring?

- Yes (1)
- No (2)

Q17 Was the option of sealing over non-cavitated decay included in your dental school training?

- Yes (1)
- No (2)

Q18 Sealants are very effective in arresting decay when there are noncavitated occlusal caries.

- Strongly agree (1)
- Somewhat agree (2)
- Neither agree nor disagree (3)
- Somewhat disagree (4)
- Strongly disagree (5)

Q19 Do you think restoring a noncavitated occlusal carious lesion provides a better outcome for the patient when compared with a sealant?

- Always (1)
- Most of the time (2)
- About half the time (3)
- Sometimes (4)
- Never (5)

Q20 Do you think that placing sealants to arrest the progression of noncavitated carious lesions is within the standard of care?

- Yes (1)
- Maybe (2)
- No (3)

Q21 Placing sealants to arrest the progression of noncavitated carious lesions would put me at risk from a liability perspective.

- Strongly agree (1)
- Somewhat agree (2)
- Neither agree nor disagree (3)
- Somewhat disagree (4)
- Strongly disagree (5)

Q22 Guidelines, Guidance, and What Other Dentists are Doing

This next set of questions asks about your familiarity with guidelines, guidance, and what other dentists are doing.

Q23 What percentage of your PDA colleagues do you believe routinely apply sealants to noncavitated occlusal carious lesions?

- 0 - 5% (1)
- 6 - 25% (2)
- 26 - 50% (3)
- More than 50% (4)

Q24 Are you aware of any guidelines published by a professional dental organization or society (outside of Permanente Dental Associates) regarding the use of sealants for the prevention of caries?

- Yes (1)
- No (2)

Q25 Are you aware of any guidelines published by a professional dental organization or society (outside of Permanente Dental Associates) regarding the use of sealants to manage noncavitated occlusal carious lesions?

- Yes (1)
- No (2)

Q26 Are you aware of any guidance/policy from the Permanente Dental Associates (PDA) Clinical Effectiveness Committee regarding how noncavitated occlusal carious lesions should be managed?

- Yes (1)
- No (2)

If No Is Selected, Then Skip To Do you think the PDA Clinical Effecti...If No Is Selected, Then Skip To Do you think the PDA Clinical Effecti...

Q27 Do you think having guidance/policy from the PDA Clinical Effectiveness Committee on managing noncavitated occlusal carious lesions is a good thing?

- Yes (1)
- Maybe (2)
- No (3)

If Yes Is Selected, Then Skip To Do you think the PDA Clinical Effecti...If Maybe Is Selected, Then Skip To Do you think the PDA Clinical Effecti...If No Is Selected, Then Skip To Do you think the PDA Clinical Effecti...

Q28 Do you think the PDA Clinical Effectiveness Committee should provide guidance/policy regarding how noncavitated occlusal carious lesions should be managed?

- Yes (1)
- I'm not sure/need more information (2)
- No (3)

Q29 Do you think the PDA Clinical Effectiveness Committee should establish clinical performance standards regarding how noncavitated occlusal carious lesions should be managed?

- Yes (1)
- I'm not sure/need more information (2)
- No (3)

Q30 Do you think the PDA Clinical Effectiveness Committee should conduct audits and provide individual performance feedback regarding how you manage noncavitated occlusal carious lesions?

- Yes (1)
- I'm not sure/need more information (2)
- No (3)

Q31 Diagnostic Codes, Workflow, and Salary

This next set of questions asks about your familiarity with diagnostic codes, your opinions about the workflow in your clinic, and how your salary is established.

Q32 Have you heard about the development of diagnostic codes in dentistry?

- Yes (1)
- No (2)

Q33 Could hygiene and assistant staff do an adequate job placing a sealant over a noncavitated occlusal carious lesion?

- Yes (1)
- Sometimes, under specific circumstances (2)
- No (3)

Q34 Does the hygiene and assistant staff have capacity in their schedules to treat patients who need sealants for noncavitated occlusal carious lesions?

- Yes (1)
- Sometimes (2)
- No (3)

Q35 How easy would it be to change the workflow in your clinic to allow the dental hygienist and dentist to routinely apply sealants on noncavitated occlusal carious lesions?

- Extremely easy (1)
- Somewhat easy (2)
- Neither easy nor difficult (3)
- Somewhat difficult (4)
- Extremely difficult (5)

Q36 Is part of your salary based on quality measures regarding how you manage your patients?

- Yes (1)
- Maybe (2)
- No (3)

Answer If Is part of your salary based on quality measures regarding how you manage your patients? Yes Is Selected

Q37 What percentage of your salary should be based on how you manage your patients?

- 0% (1)
- 1 - 5% (2)
- 6 - 15% (3)
- 16 - 25% (4)
- More than 25% (5)

Q38 How many patients do you see in an average week?

- 1 - 25 (1)
- 26 - 50 (2)
- 51 - 75 (3)
- 76 - 100 (4)
- More than 100 (5)

Q39 In what year did you graduate from dental school?
